# Suicidal behaviors and associated factors among patients attending an emergency department: a facility-based cross-sectional study

**DOI:** 10.1186/s12888-023-04949-9

**Published:** 2023-06-25

**Authors:** Dureti Kassim Wordefo, Faiz Mohammed Kassim, Elizabeth Birhanu, Girma Mamo

**Affiliations:** Department of Psychiatry, St. Paul’s Hospital Millennium Medical College, Addis Ababa, Ethiopia

**Keywords:** Emergency department, Suicide, Suicidal behavior, Suicide attempt, Suicide risk

## Abstract

**Background:**

Emergency departments (ED) are an important site for screening patients with suicidal behaviors. However, there is insufficient data in low-and middle-income countries regarding the magnitude of suicidal attempts among patients attending EDs. Therefore, the present study aimed to screen suicidal behavior and factors associated with suicide in patients attending an ED of Addis Ababa Burn, Emergency and Trauma Hospital, Ethiopia.

**Method:**

A facility-based quantitative cross-sectional study was conducted between April and June 2018. A total of 398 participants were recruited using a consecutive sampling technique. The collected data collected includes structured questionnaires containing sociodemographic determinants, chronic medical illness conditions, substance use characteristics, social support level, common mental disorders (CMD) screening, suicidal behaviors assessment and suicidal attempts reason and method.

**Results:**

The prevalence of suicidal behavior and suicidal attempts were 8% and 6.3%, respectively. Suicide was attempted most frequently in the 18–24 age group. There was no overall difference in sex distribution for suicidal attempts. However, there were sex-based differences when the age group was taken into consideration. The commonest underlying reason for the attempt was social reasons (44%), while the most frequently reported attempt method was hanging (36%). No single factor was found to be significantly associated with the suicidal attempt.

**Conclusion:**

Although suicidal behaviors are more common in patients attending the ED than in the general population, these facts have previously got little attention in patient attending EDs in low and middle income countries. The present findings support the need for a more detailed assessment of suicidal behaviours in patients attending ED and in patients with CMD.

**Supplementary Information:**

The online version contains supplementary material available at 10.1186/s12888-023-04949-9.

## Introduction

Suicide represents a huge global health concern, with nearly 800,000 people dying every year [1]. Suicidal behaviors are also classified among the leading causes of death and injuries that involve a wide range of age group in several countries [2, 3]. In many countries, the rate of suicidal attempt is unknown, despite a concern since the end of the last century that suicide is estimated to be 10–20 times higher than the official data [4–7].

Various “ideation to action” theories have been proposed to describe suicidal behavior [8–10]. Some studies use suicidal thought or ideation, plan and attempt to describe suicidal behavior, which is a general encompassing term [5, 11–13]. Suicidal ideations is also a broad nomenclature that is used to explain a range of thoughts, preoccupations, ideas, and contemplations about suicide and death [14]. On the subject matter, there is an argument that it is better to use suicide attempts for any kinds of suicide-related gestures or actions, although some use the term “suicide gesture” to describe activities before the attempt and to differentiate from suicide attempts themselves [15]. It is generally agreed that suicide ideation or contemplation is a precondition for suicide-related actions or attempts [10] and around 30% of people with suicidal ideation commonly attempt suicide [16]. Therefore, it is expected that suicidal behavior is a more frequent problem than suicide per se.

Suicide, or a suicide attempt, is considered as a multi-factorial behavior, and is associated with multiple risk factors that needs a “network analysis” of psychological, sociological, clinical, biological and cognitive factors [17–19]. The majority of the risk factors are identifiable, allowing the anticipation of suicidal attempts and the opportunity to apply appropriate interventions to prevent suicidal behavior [20–23]. However, there are no precise clinical tools or gold standards that provide a predictive value to deal with suicide identification and intervention, which makes employing suicide prevention programs challenging [14, 24]. Studies suggested that collaboration between emergency departments (EDs) and mental health services [25, 26] and improving suicide risk assessment tools in EDs enhances suicide prevention [14, 27–30].

Psychiatric disorders are key risk factors for suicide in Ethiopia [31–34] and other countries [12, 13, 35–45]. However, psychiatric disorders alone do not sufficiently explain suicide [22]. Other important factors include childhood maltreatment [46], alcohol or substance use [13, 35, 47–50], chronic medical conditions, socio-economic status, family dysfunction and lack of social support [15, 38], or demographic factors such as gender [21], sexual abuse [42, 51, 52] or some personality traits such as perfectionism, high impulsivity, high openness, neuroticism, or low self-esteem [12, 23, 50, 53]. Factors such as hopelessness and pain are often essential triggers of suicidal ideation [8].

Although suicide is a global problem, the degree of risk factors may not be universal and can change with circumstances, cultures or even inter-facilities in the same country [5–7]. The interplay between a number of risk and protective factors at individual and psychosocial levels must be taken into consideration when attempting to understand which factors promote resiliency, or alternatively, vulnerability to suicide and suicidal behavior [54]. Furthermore, while suicide is a serious problem in high-income countries that had the highest age-standardized suicide rate, 79% of suicides worldwide occurred in low- and middle income countries [1]. Although suicidal behavior remains an important health problem in the african countries, studies about suicide and associated risk factors across the continent are limited, [see 7, 55 for review]. Likewise, epidemiological studies indicated that the magnitude of suicide ideation and attempts are high in the Ethiopian general population [for review, see 56]. According to a WHO report, around 7,000 people (5700 male) committed suicide in Ethiopia in 2016, with a rate of 7.2 per 100,000 persons per year [1]. However, there are limited number of facility or population based studies on suicide and suicidal behaviors in Ethiopia [31–34, 55–65].

To the best of our knowledge, there are no studies about suicide and suicidal behaviors among patients attending ED in Ethiopia, although EDs are crucial sites to assess patients with suicidal behaviors [14, 27, 30, 66–72]. Therefore, the present study aimed to screen the prevalence of suicidal behavior and suicidal attempt, and to identify risk factors associated with suicide in patients attending ED of Addis Ababa Burn, Emergency and Trauma hospital (AABET) either for medical or surgical emergency conditions. We predicted that the prevalence of suicidal behavior would be between 10 and 30% in patients attending ED, based on previous facility and community based studies. We also predicted that there would be identifiable risk factors which predisposes individuals to attempt suicide.

## Materials and methods

The study participants were enrolled in a facility-based quantitative cross-sectional study to assess the prevalence of suicidal attempt among patients 18 years and above attending AABET from April to June 2018. AABET hospital is an affiliated hospital of Saint Paul’s Hospital Millennium Medical College (SPHMMC), which is one of the two teritiary specialized hospitals in Ethiopia.

Ethical approval and clearance was obtained from Institutional Review Board of St. Paul’s Hospital Millennium Medical College. After detail explanation about the study before the data collection, an informed consent was obtained from the study participant or, if the participants were illiterates, from a legal guardian. They were also informed that they can withdraw from the study at any time they want. If participants experienced adverse events, arrangements were made with the hospital if there was a need of emergency care for the participants.

### Study subjects

Participants were all patients who visited the ED of AABET hospital either for medical or surgical emergency conditions during the data collection period and eligible to participate in the study according to the inclusion and exclusion criteria.

### Inclusion criteria

Adults above the age of 18 years attending the ED who are willing to participate in the study. Subjects who were severely and/or acutely ill at the time of data collection were excluded, as well as those who have cognitive impairments the medical illness severity.

### Sample size determination and sampling procedure

#### Sample size determination

The sample size was calculated using formula for estimating single population proportion employing expected prevalence of suicidal attempt among patients seen in ED to be 50% as there was no prior study on the subject in the EDs (However,  it can also be 10–30% based on the average between general population and people with mental illnesses in Ethiopia), at 95% confidence interval and 5% margin of error giving 384. A non-response rate or drop-out of 10% was used to give a final sample size of 423.

#### Sampling procedure

All patients that visited AABET hospital ED during the data collection period and fulfilled the eligibility criteria were included in the study by using consecutive sampling technique until the required sample size was achieved.

### Data collection tools and procedure

#### Data collection tools

The questionnaire was developed based on sources from previous studies with some modification to fit into our setup. The questionnaire was translated from English into Amharic and Afan Oromo languages by native speakers of the languages who are also fluent in English and then back-translated into English by other translators to check its consistency in translation. The questionnaire was pretested and had seven subsections; (i) socio-demography, (ii) chronic medical illness related, (iii) substance related, (iv)mental health related, (v) social support, (vi) questions related to suicidal behaviors and (vii) reasons and means of suicidal attempts. Participants were screened for substance use using Alcohol, Smoking and Substance Involvement Screening Test (ASSIST) questionnaire. The Oslo 3-items social support scale was used to measure the strength of social support. The Suicidal Behaviors Questionnaire revised (SBQ-R) was used to assess for suicidal behaviors. The other questionnaires were developed by the investigator from different literatures.

The Kessler Psychological Distress Scale (K10) is used to assess nonspecific or common mental disorders (CMD) such as anxiety and depression, and is widely applied to differentiate people with CMD from those without a mental disorder [73, 74]. K10 was validated and widely used in Ethiopia [75–77]. These questions addresses how the participants have been feeling over the past one month. Some of the included questions include “During the last 30 days, about how often did you feel tired out for no good reason?” and “During the last 30 days, about how often did you feel hopeless?” All items are answered on a 5-point Likert type scale: 0 = none of the time; 1 = a little of the time; 2 = some of the time; 3 = most of the time; 4 = all of the time….” The participants were said to have CMD. if they scored ≥ 7 on Kessler-10 scale.,

#### Data collection method

The data were collected by trained clinical and psychiatry nurses using a face to face interview method.

#### Data analysis

Data was entered using Epi Info 7 and transferred to SPSS version 20.0 software for analysis. Proportions, percentages, ratios, frequency distribution, measures of central tendency and measures of dispersion were used to describe the data on the prevalence of suicidal attempt. Chi square test was used to see the association between suicidal attempts with different independent variables including Socio-Demographic variables. Multivariable analysis was done using binary logistic regression to control confounders and identify factors with significant association. Factors with P-value less than 0.2 during the bivariate analysis was entered in the final model for the multivariable analysis. Adjusted Odds Ratios with 95% Confidence Interval and P-values less than 0.05 were used to determine significant association.

#### Independent variables

Sociodemographic characteristics, suicidal attempt history, physical and mental health, social factors, and biological factors.

#### Dependent variable

Suicidal behavior, suicidal ideation and suicidal attempt.

#### Operational definition

[Supplementary 1].

## Results

### Sociodemographic characteristics

A total of 398 willing to participants (18–97 years of age) were included, with a response rate of 94.1%. The details of the demographic results are shown in Table 1.

**Table 1:** Sociodemographic characteristics of study subjects (n = 398)

Characteristics	Number	Percent (%)
**Sex**		
Male	240	60.3
Female	158	39.7
**Age(years)**		
18–27	108	27.1
28–37	126	31.7
38–47	79	19.8
48–57	43	10.8
58–67	32	8.0
≥68	10	2.5
**Marital status (395)**		
Married	214	54.2
Single	129	32.7
Divorced	30	7.6
Widowed	13	3.3
Unmarried couples	9	2.3
**Religion (382)**		
Orthodox	257	67.3
Muslim	63	16.5
Protestant	56	14.7
Catholic	5	1.3
Others	1	0.3
**Educational status (n = 393)**		
College and above	103	26.2
7-12^th^grade	135	34.4
1-6^th^grade	78	19.8
Read and write	51	12.9
Illiterate	26	6.6
**Occupation (n = 395)**		
Civil servant	75	19.0
Merchant	69	17.5
House wife	64	16.2
Farmer	55	13.9
Student	47	11.9
Daily laborer	42	10.6
No job	28	7.1
Other jobs^o^	15	3.8

^o^: Other jobs includes retired, house servant, preacher, driver, driver assistant)

**Table 2:** Substance Involvement Scores of study subjects (n = 398)

Characteristics	Number	Percent
**Tobacco use**		
Lower risk	379	95.2
Moderate risk	14	3.5
High risk	5	1.3
**Alcohol use**		
Lower risk	383	96.2
Moderate risk	6	1.5
High risk	9	2.3
**Khat use**		
Lower risk	371	93.2
Moderate risk	15	3.8
High risk	12	3.0
**Cannabis use**		
Lower risk	395	99.2
Moderate risk	2	0.5
High risk	1	0.3

### Chronic medical illness

Around 19% participants (76) had chronic medical illness: hypertension (28.9%), diabetes mellitus (25.0%), HIV (13.2%), and other illness such as epilepsy or stroke (13.1%). Around 20% (15) did not know the type of illness they have.

### Substance use history

Substance use history results showed that out of 398 participants, 53 (13.3%), 42 (10.6%), 28 (7%) and 11 (2.8%) had ever used alcoholic beverages, khat, tobacco, and cannabis, respectively. Furthermore, to examine the risk of substance use, study participants were screened using ASSIST score, and risk score for each substance used was determined. The score obtained for alcohol (or other substance) will be categorized under “high risk”, “moderate risk” and “low risk”, if the study participant scores ≥ 27, 11–26 (or 4–26 for other substance), and 0–10 (or 0–3 for other substance), respectively. Subsequently, as shown in Table 2, 3% were in the high risk for khat, 2.3% were in the high risk for alcoholic drinks, 1.3% were in the high risk for tobacco, and 0.3% were in the high risk for cannabis.

### Common mental disorders (CMD)

The results showed that, out of the 398 participants, more than one third (174 or 43.7%) of them were found to have CMD. From those with CMD (174), 81 (46.6%) were female. The results also revealed that suicidal behavior among patients with CMD was 16.9%, while it was 1.3% in patients without CMD. In addition, suicidal attempt among patients with CMD was 14.4%. In other words, 100% of those who attempted suicide (25) were from CMD group.

### Social support

**Fig. 1:**
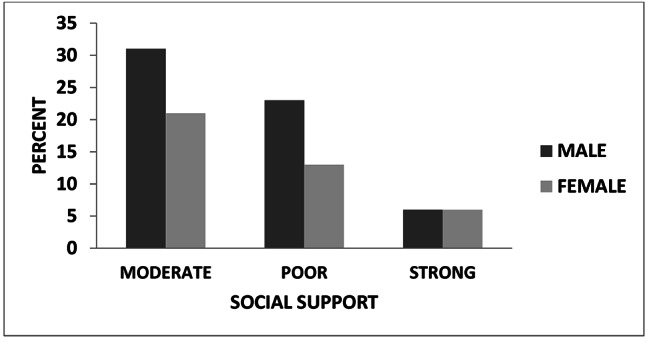
Social support status of study participants (n = 394)

The participants was categorized under poor, moderate or strong social support if they scored 3–8, 9–11 or 12–14 on Oslo 3 social support scale, respectively. Out of them, 11.6% had strong, 52.8% had moderate, and the remaining (37.6%) had poor social support. As shown in Fig. 1, the proportion of males is greater in the poor and moderate social support group.

### Suicidal-related assessment

**Fig. 2:**
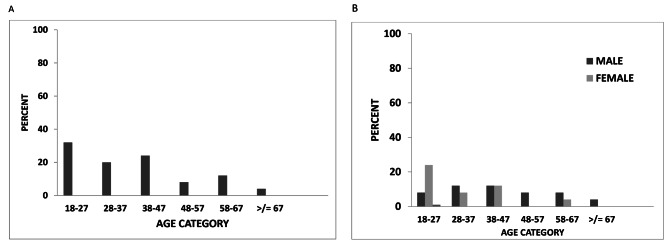
Proportion of suicidal attempt among study participants based on age (**A**) and based on sex and age distribution (**B**) (n = 394)

8% (32) of the participants had suicidal behaviors: the magnitude of life time suicidal attempt was 6.3% (25), while the remaining 7 (1.7%) had suicidal ideation. The majority (64%) had history of suicidal attempt only once, while 28% (7) of them had twice and the remaining 8% (2) had three or more times. Furthermore, the proportion of those with current suicide behavior and responded that they likely will attempt suicide in the future are 1.8%. As shown in Fig. 2A, 32% (10) of those with suicidal attempt lies in the age category 18–27 years, followed by the age category 38–47 (24%). Overall, the female and male distribution in the history of suicidal attempt was nearly equal (1:1.1). However, as presented in Fig. 2B, a sex and age distribution of the study participants showed that there is a sex-based distribution difference for a specific age category. For example, in the age category 18–27, females are greater in proportion, while in the age category 28–37 males are greater.

**Table 3:** Details of suicidal attempts (n=25) done by study participants

Characteristics	Number	Percent
**Methods of suicidal attempt**		
Hanging	9	36
Poisoning		
Non-medication (pesticides) poisoning	5	20
Medication Poisoning	3	12
Electricity	5	20
Alcohol intoxication	1	4
Gun shot	1	4
Vehicle injury	1	4
**Reasons for suicidal attempt**		
Social problems	11	44
Mental illness related	6	24
Non-psychiatric medical illness related	4	16
Demotion from work	1	4
Alcohol intoxication	1	4
Financial problem	1	4
I don’t know	1	4

### Suicidal attempt reason and method

Suicidal attempt due to social reasons (such as like divorce or conflict) took the highest proportion (44%), followed by mental illness (24%) and non-psychiatric medical illness (16%). As shown in Table 3, hanging, medical and non-medical poisoning and electricity were the major methods of attempting suicide. 60% of the participants were happy that they have survived, while 16% reported they felt guilty about attempting to end their life. However, 12% were angry for the unsuccessful attempt, but the remaining 12% were indifferent. Furthermore, family history of suicide was found in 2 (8.0%) of those with history of suicidal attempt, while 18 (72%) of them did not have family history of suicide.

Finally, multivariable analysis was done using binary logistic regression to control confounders and identify factors with significant associations. Social support (COR = 0.52, 95% CI 0.23–1.02, p = 0.06), occupation (COR = 0.87, 95% CI 0.72–1.06 and P = 0.1) and alcohol risk level (COR = 1.91, 95% CI 0.83–1.06 and P = 0.13) showed a crude association with suicidal attempts during bivariate analysis. However, when factors were entered in the final model for the multivariable analysis no significant association was found between the factors and suicide attempt.

## Discussion

The main objective of the present study was to assess the magnitude of suicidal behaviors and the possible factors associated with suicidal behaviors among patients attending ED of AABET. To the best of our knowledge, this is the first study to screen suicidal behavior and/or suicide attempts in Ethiopian patients who visit the ED. The results showed that the prevalence of suicidal attempt was 6.3% and the rate of suicidal behavior was 8%. There were even sex distribution for suicidal attempts in the overall participants. Suicide was attempted most frequently in the age group 18–24. The common underlying reason for the attempt was social reasons, while the most frequently reported attempt method was hanging. The majority (60%) reported that they feel happy for surviving. No factor was found to be significantly associated with suicidal attempt.

We have screened that 43% the participants had CMDs, which is comparable with a recent large scale study report that based on several national registers [44]. A meta-analysis study found that a psychiatric-related illnesses were around 85% among patients with suicidal behaviors in EDs [43]. Likewise, another meta-analysis study on the prevalence of a psychiatric-related illnesses at EDs reported that 4% of the attendants were because of mental-illnesses, but around 58% of attendants were identified with a mental illness history [78]. In contrast, our finding is higher than other studies that reported 30–36% depression or mood disorders but these studies assessed only affective disorders [79–81]. Regardless of the magnitude variations across different nations, results show that mental-illnesses are a common problem among patients attending EDs. However, most patients with suicidal behaviors at the EDs are discharged without receiving assessments for common mental-illnesses [30, 82].

The present study detected that suicidal behavior among patients attending ED is 8%, which is comparable with previous finding (10%) among patients attending a primary care facilities in five low-and middle income countries, including Ethiopia [7]. However their report [7] indicates that the overall prevalence of suicide attempt (2.2%) was lower than our finding. Moreover, the present suicidal behavior magnitude is in the range of prevalence that Jordans, Rathod [7] found in five countries: suicidal ideation in facility-based samples was 5.0-14.8%, while it was 3.5–11.1% in community-based samples. Our finding is also consistent with previous suicidal behavior reports in the USA [83, 84] but slightly higher than most multicenter-based findings (3–6%) in the USA [35, 85, 86] and China [87]. However, our finding is lower than the report (12%) of the same team [81] and other reports (12.8%) among patients in the EDs in the USA [80], indicating that suicidal behaviors vary across nations.

Furthermore, the magnitude of suicidal attempt in Ethiopia is not consistent [31–34, 56–63]. The present results showed that the prevalence of suicidal attempt in the ED is 6.3% which is higher than the global lifetime prevalence rates of 2.7% [88] and some previous reports in the Ethiopia general population that ranges between 0.9 and 4.4% [58–60, 63]. Other studies in Ethiopia reported that a suicidal attempt among psychiatric patients [31–34, 59] and epileptic patients [61] ranges between 4.1 and 51.3%, which is higher than the findings in the general population and the present finding. However, the prevalence obsereved in our study is generally in agreement with previous reports about overall suicidal attempt in the Ethiopian general population that ranges between 1.4 and 19% [for review, see 56]. Altogether, the present result shows that the magnitude of a suicidal attempt among patients in the ED lies between previous reports about suicidal attempt in the general population and patients with psychiatric disorders. This could be explained by the fact that our study was done in emergency patients who are at high risk as compared to the general population found in the community, but at a lesser risk than patients with severe mental illness.

Interestingly, the sex distribution of the suicidal attempt in the present study varies based on the age category, which is comparable with sex and gender based suicidal behavior in South Africa [89]. In the present study, females predominate in attempt distribution for the 18–27 age category, but males predominate for the 28–37 age category, and both sexes are similar in the 38–47 age category. However, regardless of age category, the overall sex distribution of suicidal attempt is nearly equal for both sexes, which is comparable with previous studies in Ethiopia [55] and at the ED in the UK [26]. Likewise, Kebede and Alem [64] found that magnitude of suicidal attempt and ideation among adults in Addis Ababa are similar for female and male, although female sex and younger age group is associated with suicidal ideation. A previous study in our facility that focused on suicidal behaviour among outpatient adults found that there was no significant difference in suicidal attempt by sex, although females had significant predominance in suicidal behaviours than males [59]. There was no association between suicidal behaviour and gender among high-school adolescents [64]. Alem, Kebede (61) also found that the sex distribution for suicidal attempt among adults in Butajira/Ethiopia is nearly equal. On the contrary, a study conducted in Gambella/Ethiopia reported that more female youths in the age group of 20–28 attempted and completed suicide [58]. The findings for sex-based distribution in suicidal attempt in Africa is mixed, some reporting equal ratio or higher prevalence in either male or females [see 55 for review], which is comparable with reports from the European multi-center study some decades ago [6]. On the other hand, the evidence from Italy showed that females have predominance in attempting suicide [13]. Jordans, Rathod [7] and colleagues also found that the overall suicidal behavior is associated with being female in five low and middle income countries. Another study in South Africa found that being female was associated with suicidal behavior, although the suicidal attempt was nearly equal among both sexes [89]. Moreover, among adults with psychiatric disorders in Gonder/Ethiopia, Mekonnen and Kebede [31] found that more females attempted suicide than males, although suicide ideation is similar for both sexes. There were no sex differences in suicidal behavior among adults with psychiatric disorders in Jimma/Ethiopia [32]. Likewise, Shibre, Hanlon [33] reported that there were no sex differences in suicidal attempt among people with psychiatric disorders in Butajira, although completed suicide was more common in males. The equal prevalence between females and males in most studies in Ethiopia could be because of the belief in society that “suicide is a cursed act” that makes females more conservative in disclosing about their suicidal behavior. These results overall suggest that although males and females are equal suicide ideators and attempters in the general population, females in the younger age group need special attention to prevent suicidal behavior.

In our study, the most common age of suicidal attempt was 18–24 years, which is in agreement with previous community-based [60, 63] and facility-based studies in Ethiopia [32]. Importantly, Kebede and Ketsela [57] reported that suicidal attempts in adolescents in Addis Ababa high schools is 14.3% that is higher than studies in the general population, which may indicate that the adolescent age group is at higher risk. Likewise, a community-based study among high school students found that suicide attempt is around 16% [64]. Studies in different countries, including South Korea, USA, Jamaica and Spain, reported that the suicidal attempt rate in late school-aged children and adolescents is around 4–25% [16, 36, 42, 90–92]. For example, a study in the USA reported that the rate of suicidal attempt in adolescent is 7.4% [28], but the risk of suicide is higher (18.5%). Scocco, de Girolamo [13] also reported that although the overall rate of suicidal behavior (i.e., 4%) or attempt (i.e., 0.5%) was lower in Italy relative to other European countries, there was increased risk of suicide ideation among students (OR, 4.0). Whittier, Gelaye [60] also found that suicidal behaviour is most common in middle-aged adult outpatients in Ethiopia. A cohort study conducted in Butajira by Shibre, Hanlon [33] found that being ≥ 40 years was inversely correlated with suicidal attempt in people with mental illness, similarly indicating that younger age is a risk factor for suicidal behaviour. Alem also showed that there seems to be a decline in the frequency of suicidal attempt with increasing age [60], which is consistent with the finding in Italy [13]. A facility-based study in South Africa reported that suicidal behavior is inversely associated with age [89]. Likewise, a study in the USA reported that suicidal behavior declined with age among older patients attending an ED [93], although there are contrasting reports regarding the association of age and suicide [53]. There are several factors that might trigger adolescents or youths around 20 years old to have suicidal behavior. Some of the important factors include drug or alcohol abuse, psychotic disorders, smoking, a history of sexual abuse, severe medical conditions, and parental issue [52, 90]. Previous suicide attempt history is also associated with current suicide attempt among adolescents, compared to adults,in the emergency departments [74]. Overall, the present result supports previous findings that the younger adults are more vulnerable to suicidal behavior.

Regarding method of suicidal attempt, the present result showed that the predominant methods were hanging and poisoning which is in agreement with previous facility or community-based studies in Ethiopia [31–34, 58, 60, 61, 63, 94]. Likewise, the most common methods of suicidal attempt in African countries are hanging and poisoning [95]. In our study, intentional pesticides use rather than medication-overdose was the most common method of poisoning next to hanging, which is also comparable with other studies in Ethiopia [32, 33] and Africa [96]. Our finding is also consistent with the global suicide rate (14–20%) because of pesticide self-poisoning [97]. A previous study at a specialized hospital in Ethiopia also showed that pesticide poisoning most frequently applied as a suicidal attempt method, suggesting that pesticide-use control method is nationally required [98]. The other important finding in the current study is that electricity was as common as pesticide-based poisoning method, although it was rarely reported in previous studies [31, 94]. Overall, although it is easy to identify the common suicide attempt methods, the intervention program may not be as easy as a policy implications for pesticide control mechanisms. A “one-size-fits-all” approach to reduce or to prevent suicidal behaviour will not be effective [99]. Therefore, holistic approaches starting from mental-health assessments in the EDs and exploring the psychopathology of suicide to multiple clinical treatment options warrant.

The majority (44%) of participants in our study stated that their underlying reason to attempt suicide was social problems (such as divorce or conflict with other people) which is comparable with other community-based [60] and institution-based studies in Ethiopia [61]. Our finding also showed that mental illness and other medical conditions are the second and third most common reasons to attempt suicide, respectively. We also found that all (100%) of patients who attempted suicide were with CMD, and 97.7% of suicidal behaviours were among patients with CMD. Mekonnen and Kebede [31] reported that social reason (21%) was a second predominant factor next to medical illness (65.1%) to attempt suicide. The main reason for the discrepancy between Mekonnen and Kebede [31] and our result might be due to that they conducted their studies among adults with psychiatric disorders, while we conducted among adults in the ED. Although poor social support, occupation status, and alcohol use level showed crude association with suicide attempt, multivariate analysis failed to show significant association between one of those factors or other factors (such as sex, age, substance use history and mental illness) and suicidal attempt. Likewise, other community or facility-based studies in Ethiopia did not find associations between sex, ethnicity, or religion and suicidal attempt [63] or between suicidal attempt and sex, marital status, educational level, religion, family history of suicide or type of mental illness [31]. In contrast, facility-based studies conducted in Jimma [32] and Mekelle [34] found that factors such as major depressive disorder, other mental illnesses or family history of mental illness are associated with suicidal behaviour among people with psychiatric disorders, which are consistent with reports from other countries [38, 39]. In addition, alcohol use disorder and nicotine dependence are associated with suicidal behaviour among people with mental illness in Ethiopia [32]. Another community-based study in Ethiopia found a strong association between major depressive disorder with suicidal attempts among people with severe psychiatric disorders [33]. Haile, Awoke [62] also found that poor social support, mental illness or co-morbid depression and family suicide history were associated with suicidal behaviour among patients with epilepsy. Overall, results support that mental illnesses, poor social support, and alcohol use level are important risk factors for a suicidal behavior.

We found that around one third of participants (36%) attempted suicide more than once. A study in Ethiopia reported that a previous attempts history and family history of suicide [34] are associated with suicidal behaviour among people with mental illness. Studies in several countries also showed that a prior suicidal attempt or thought [100–106] and family history of suicidal attempt [38–40, 107] were associated with an increased risk of a new suicide attempt. Importantly, some studies emphasized that a prior suicide attempt is the greatest predictor of a completed suicide [37] or a subsequent attempt [36]. A study conducted in four English speaking countries showed that the factors that have strong association with suicidal behaviour were depression, hopelessness, poor self-esteem, poor resilience, and less access to mental health services [103, 108]. However, earlier identified risk factors such as depression, hopelessness, impulsivity or most psychiatric disorders do not strongly predict suicidal attempts but suicide ideation [9]. It has been found that in addition to non-fatal self-harm [50], factors that decrease fear of death, pain or injury make the transition easier from suicidal ideation to action [for review, see 8]. In general, worldwide evidence suggests that important risk factors for suicide among adolescents or late school-aged children include mental illnesses, suicide attempt history [36], self-esteem and bullying, personality traits, family issues, and means of committing suicide [for review, see 19, 53].

Finally, the present study tried to screen the magnitude of suicidal behaviors and associated factors using standard instruments tools. It has the strength to provide valuable baseline data to initiates further studies and also to devise interventions since suicidal behavior studies among ED patients are lacking in Ethiopia. Screening for suicidal behaviors in ED patients will be an important opportunity for suicide intervention program. However, the present study has important limitations to be considered in the future studies. First, it was not free from social desirability bias as the data was collected by a face to face interview approach that might result in over-or under reporting. Second, there might be recall bias because of forgetfulness and the information they provided was not cross-checked. Third, the present study included only adult patients in the ED. Future studies need to include school- children and adolescents. Fourth, the present study focused on CMD but not subgroups of patients. Further screening of mental illness in the EDs and its association with suicidal behavior is required. Lastly, future multi-centre studies need to assess the main medical conditions of ED visiting patients to identify whether visit triggering medical conditions and suicidal behaviours are associated.

## Conclusion

Although suicidal behaviors are more common in the ED than the previously reported prevalence in the general population, it has been ignored in the EDs of middle and low income countries. The present findings support the need for more-detailed assessment of suicidal behaviours in patients attending EDs. Efforts are required to make ED an important site to assess patients with suicidal behaviors, and patients with an identified CMD may benefit from suicidality screening. Furthermore, collaboration between EDs and mental health services are required to enhance suicide prevention in Ethiopia.

## Electronic supplementary material

Below is the link to the electronic supplementary material.

Supplementary Material 1

## Data Availability

The data sets used and/or analyzed during the current study are all included in the manuscript.
