# Supplementary material for: Suicidal behaviors and associated factors among patients attending an emergency department: a facility-based cross-sectional study
Source: BMC Psychiatry. 2023 Jun 25;23:462. doi: 10.1186/s12888-023-04949-9 (PMC10290805; doi:10.1186/s12888-023-04949-9)
Supplement: Supplementary file 1 — Supplementary Material 1 [file 12888_2023_4949_MOESM1_ESM.docx]

| **Suicidal ideation** | Thinking about, feeling, considering, contemplation, or wishing to die (Harmer et al., 2022). |
| --- | --- |
| **Suicidal Attempt** | Self-injurious behavior with a nonfatal outcome accompanied by explicit or implicit evidence that the person intended to die. |
| **Suicidal behavior** | Suicide-related phenomena (suicide thought, suicide plan or suicide attempt) (Paykel et al., 1974; Scocco et al., 2008). |
| **Risk factor for suicide** | Any detectable characteristic of an individual or group of people proved to be associated with an increased probability of suicide. |
| **At Risk of suicide** | If a study subject scores 7 or above out of the total score of 18 on Suicidal Behaviors Questionnaire – Revised (SBQ-R) |

**Supplementary: Operational definitions**
